# Aconite in Dysentery

**Published:** 1882-10

**Authors:** 


### ACONITE IN DYSENTERY.

Dr. Owen reports the results of one hundred and fifty-one cases of *acute dysentery treated with aconite.* He was induced to look about for another treatment than the conventional one with ipecac, on account of the nausea which often attends the latter, and which often drives hospital patients, especially, to rebel against the repetition of the dose. Dr. Owen gave the tincture of the British pharmacopœia, which is of one-sixth the strength of Fleming's tincture. He gave one minim every hour. This would make thirty minims in twenty-four hours. Dr. Owen feels that his experience in one hundred and fifty-one cases justifies him in speaking quite positively in favor of the treatment. In his paper he gives a very good analysis of its results.—*N. Y. Med. Jour.*

